# Supplementary material for: Using moral foundations in government communication to reduce vaccine hesitancy
Source: PLoS One. 2021 Nov 9;16(11):e0259435. doi: 10.1371/journal.pone.0259435 (PMC8577733; doi:10.1371/journal.pone.0259435)
Supplement: S1 Appendix — (ZIP) [file pone.0259435.s001.zip › S1_Appendix.pdf]

S1 Appendix: Using moral foundations in government communication to reduce vaccine hesitancy

Florian Heine[1*], Ennie Wolters[1],

1 Tilburg University, Tilburg School of Economics and Management, Tilburg, The Netherlands

* f.a.heine@tilburguniversity.edu

Moral Foundations Theory

To understand moral behaviour across cultures, Graham et al. [1] develop Moral Foundations Theory (MFT), which is built upon an understanding of the affective primacy of moral judgement. Innate moral intuitions, as modelled though MFT, have helped humans to quickly solve collective action problems through fast and automatic reactions to stimuli [2]. These affective reactions precede higher-level cognitive thinking [3], endowing people with an immediate approach or avoidance intuition.

MFT is a pluralist theory of moral psychology [4], which postulates that we need multiple dimensions to represent and explain the moral domain in its entire breadth. This is opposed to *monist* philosophies of morality, which argue that all manifestations of morality can be traced back to one basic value or virtue. This could be justice [5], sensitivity to harm [6], or happiness [7]. Proponents of a *pluralist* approach, by contrast, argue that evolutionary thinking encourages pluralism and that there are many recurrent social challenges, for why there exists a plurality of moral foundations. In this spirit, MFT “draws on both cultural and evolutionary psychology to examine the psychological mechanisms that lead people and groups to hold divergent moral values and beliefs” [4].

While Haidt [8] describes MFT as innate and universally available psychological systems, this does not preclude cultural learning. Social environments like religions,

cultures or institutions are important determinants in the process of moral development. Evolution has equipped us with the ability to recognise patterns of the social world – a skill that has co-evolved with the cultural institutions and practices throughout human history [9].

Haidt and Joseph [10] originally devised five moral foundations dimensions. Table A1 presents an overview of these five original moral foundations, including the adaptive challenge they derive from, their triggers, characteristic emotions and relevant virtues. Later, Haidt [8] proposes a sixth moral foundation: *Liberty/Oppression*. This sixth moral foundation derives from the adaptive challenge of keeping tyrants, bullies and alpha males from becoming too powerful [8]. People who emphasise Liberty/Oppression as a moral foundation object to things that feel like (attempted) domination or oppression. Table A2 summarises the adaptive challenge, their triggers, characteristic emotions and relevant virtues of the moral foundation Liberty/Oppression.

Table A1. The five original Moral Foundations [8]

	**Care/Harm**	**Fairness/Cheating**	**Loyalty/Betrayal**	**Authority/Subversion**	**Purity/Degradation**
**Adaptive Challenge**	Protect and care for children	Reap benefits of two-way partnerships	Form cohesive coalitions	Forge beneficial relationships with hierarchies	Avoid containments
**Original Triggers**	Suffering, distress or neediness expressed by one's child	Cheating, cooperation, deception	Threat or challenge to group	Sign of dominance and submission	Waste products, diseased people
**Current Triggers**	Baby seals, cute cartoon characters	Marital fidelity, broken vending machines	Sports teams, nations	Bosses, respected professionals	Taboo ideas (communism, racism)
**Characteristic Emotions**	Compassion	Anger, gratitude, guilt	Group pride, rage at traitors	Respect, fear	Disgust
**Relevant Virtues**	Caring, kindness	Fairness, justice, trustworthiness	Loyalty, patriotism, self-sacrifice	Obedience, deference	Temperance, chastity, piety, cleanliness

Table A2. The sixth Moral Foundation, Liberty/Oppression [8]

	**Liberty/Oppression**
**Adaptive Challenge**	Keeping tyrants, bullies and alpha males from becoming too powerful
**Original Triggers**	(Attempted) domination or oppression
**Current Triggers**	Things that feel like domination or constraint
**Characteristic Emotions**	Reactance and resentment towards dominators
**Relevant Virtues**	Individuals' autonomy and control over their own matters

In the following we briefly describe the moral foundation dimensions, how they became part of human nature, and what triggers each dimension. Haidt [8], in particular Chapter 7, provides a much more detailed account of MFT and its dimensions.

The Care/Harm Foundation

This dimension is related to our evolution as a primate species, which invests a lot more into social attachment systems than other species do. This includes the ability to care for the harm of others and it extends beyond kinship or even the own species. Triggers for this dimension include topics of caring, nurturing, protecting, or interacting.

The Fairness/Cheating Foundation

This dimension is linked to the concept of reciprocal altruism. Altruism towards non-kin has been one of the big puzzles to evolutionary thinking until Trivers [11] published a theory of reciprocal altruism. We humans are among the species who have developed a "tit for tat" strategy in which we cooperate only with those that have been nice to us and reject/punish those that have taken advantage of us. Alternative strategies have evolutionarily proven to be less successful. For example, unconditional altruism invites exploitation, or strict selfishness can work at most once in social interactions, after which cooperation seizes to be sustainable. Related triggers are topics of fairness, cheating, rights, autonomy, equality, and proportionality.

The Loyalty/Betrayal Foundation

Humans have a long history of living in tribal clans. This dimension models this connection, which extends into coalition forming, family ties, sports teams or nations. The Loyalty/Betrayal foundation is “part of our innate preparation for meeting the adaptive challenge of forming cohesive coalitions” [8]. Triggers for this foundation are linked to the juxtaposition of team-players versus traitors, particularly in a competitive situation. Virtues of patriotism and self-sacrifice for the group is what drives this dimension.

The Authority/Subversion Foundation

The aforementioned social structure of human living comes hand in hand with hierarchical social interactions. The modules of this dimension “help individuals meet the adaptive challenge of forging beneficial relationships within hierarchies” [8]. Triggers for this include issues of leadership and followership, as well as deference to legitimate authority and respect for traditions. Authority/Subversion extends towards protecting the order and fending off chaos, partly by holding people accountable for complying with their obligations.

The Purity/Degradation Foundation

Sometimes referred to as Sanctity/Degradation in literature, this foundation has developed through the concept of disgust and contamination. Stemming from the need to avoid pathogens and parasites, this dimension also extends into aspects of attitudes towards out-group members such as immigrants. Furthermore, religious or quasi-religious (such as a love or disdain of industrialism, capitalism, or automobiles) images of sanctity are prominent triggers for this dimension.

The Liberty/Oppression Foundation

Haidt [8] proposes this as sixth dimension. This foundation concerns feelings towards dominance or a restriction of liberty, such as a disdain for bullies or dominators. Triggers for this dimension are concerns about liberty, autonomy and freedom [12].

Table A3. Example Sentences per Dimension

Dimension	Original	Translation
Care/Harm	Alleen als duidelijk is dat een vaccin werkt en *veilig* is, mag het gebruikt worden.	Only if it is clear that a vaccine works and is *safe*, it may be used.
Fairness/Cheating	–	–
Loyalty/Betrayal	Ieder *land* heeft een eigen vaccinatieschema.	Each *nation* has its own vaccination schedule.
Authority/Subversion	Bij medicijnen voor kinderen is deze *controle* zelfs nog strenger.	For medicines for children, this *control* is even stricter.
Purity/Degradation	We vinden het belangrijk dat kinderen een *gezonde* start in het leven krijgen.	We believe it is important that children get a *wholesome* start in life.
Liberty/Oppression	Met die *waarden* willen Inge en haar man Rogier hun drie kinderen (...) grootbrengen.	It is with these *values* that Inge and her husband Rogier want to raise their three children (...).

All example sentences are from the brochure “Vaccinaties voor kinderen van 9 jaar 2019”.

Absolute Effects

To assess the effect per signal word, we adapt the analysis from Table 5 towards using an absolute word count per MFT dimension. For this, consider Model 1, now with the absolute word count per dimension for each brochure as the dimension score. We do not include the regressions with additional controls in this analysis as it is dubious how the degree of language difficulty and the share of punctuation would translate into a word count metric.

References

1. Graham J, Nosek BA, Haidt J, Iyer R, Koleva S, Ditto PH. Mapping the moral domain. Journal of Personality and Social Psychology. 2011;101(2):366.
2. Haidt J. The new synthesis in moral psychology. Science. 2007;316(5827):998–1002.
3. Zajonc RB. Feeling and thinking: Preferences need no inferences. American Psychologist. 1980;35(2):151.

Table A4. Results of OLS fixed-effects models regressing the vaccination rate for vaccinations of the Dutch NIP on the *absolute word count of* MFT dimension loading from the associated information brochures and controls. We analyse this for data reported at the national, regional and municipal level.

	(1a)	(3a)	(5a)
	**National**	**Regional**	**Municipal**
VARIABLES	Vaccination Rate		
Purity/Degradation	0.101	0.098**	0.109***
	(0.07)	(0.04)	(0.01)
Harm/Care	0.109	0.083***	0.069***
	(0.07)	(0.02)	(0.01)
Liberty/Oppression	0.268*	0.290***	0.285***
	(0.15)	(0.04)	(0.01)
Loyalty/Betrayal	−0.757*	−0.648***	−0.607***
	(0.41)	(0.13)	(0.04)
Authority/Subversion	1.055***	1.036***	1.060***
	(0.22)	(0.12)	(0.05)
Time	−0.421***	−0.535***	−0.537***
	(0.14)	(0.04)	(0.01)
Population	0.000*	0.001**	0.002***
	(0.00)	(0.00)	(0.00)
Constant	63.361***	80.653***	85.859***
	(14.18)	(3.45)	(0.49)
Number of observations	82	2050	28946
Number of panels	10	250	3530
Within model R-squared	0.588	0.487	0.267
Between model R-squared	0.305	0.268	0.514
Overall R-squared	0.153	0.123	0.273

* $p < 0.10$, ** $p < 0.05$, *** $p < 0.01$
Clustered standard errors in parentheses.

4. Graham J, Haidt J, Koleva S, Motyl M, Iyer R, Wojcik SP, et al. Moral foundations theory: The pragmatic validity of moral pluralism. In: Advances in experimental social psychology. vol. 47. Elsevier; 2013. p. 55–130.

5. Kohlberg L. From is to out: How to commit the naturalistic fallacy and get away with it in the study of moral development. Cognitive Development and Epistemology. 1971;.

6. Gray K, Young L, Waytz A. Mind perception is the essence of morality. Psychological Inquiry. 2012;23(2):101–124.

7. Harris S. The moral landscape: How science can determine human values. Simon and Schuster; 2011.

8. Haidt J. The righteous mind: Why good people are divided by politics and religion. Vintage; 2012.
9. Gifford A. Peter J. Richerson and Robert Boyd, not by genes alone: how culture transformed human evolution; 2008.
10. Haidt J, Joseph C. Intuitive ethics: How innately prepared intuitions generate culturally variable virtues. Daedalus. 2004;133(4):55–66.
11. Trivers RL. The evolution of reciprocal altruism. The Quarterly Review of Biology. 1971;46(1):35–57.
12. Iyer R, Koleva S, Graham J, Ditto P, Haidt J. Understanding libertarian morality: The psychological dispositions of self-identified libertarians. PloS ONE. 2012;7(8):e42366.

References

1. Graham J, Nosek BA, Haidt J, Iyer R, Koleva S, Ditto PH. Mapping the moral domain. Journal of Personality and Social Psychology. 2011;101(2):366.
2. Haidt J. The new synthesis in moral psychology. Science. 2007;316(5827):998–1002.
3. Zajonc RB. Feeling and thinking: Preferences need no inferences. American Psychologist. 1980;35(2):151.
4. Graham J, Haidt J, Koleva S, Motyl M, Iyer R, Wojcik SP, et al. Moral foundations theory: The pragmatic validity of moral pluralism. In: Advances in experimental social psychology. vol. 47. Elsevier; 2013. p. 55–130.
5. Kohlberg L. From is to out: How to commit the naturalistic fallacy and get away with it in the study of moral development. Cognitive Development and Epistemology. 1971;.
6. Gray K, Young L, Waytz A. Mind perception is the essence of morality. Psychological Inquiry. 2012;23(2):101–124.

7. Harris S. The moral landscape: How science can determine human values. Simon and Schuster; 2011.
8. Haidt J. The righteous mind: Why good people are divided by politics and religion. Vintage; 2012.
9. Gifford A. Peter J. Richerson and Robert Boyd, not by genes alone: how culture transformed human evolution; 2008.
10. Haidt J, Joseph C. Intuitive ethics: How innately prepared intuitions generate culturally variable virtues. Daedalus. 2004;133(4):55–66.
11. Trivers RL. The evolution of reciprocal altruism. The Quarterly Review of Biology. 1971;46(1):35–57.
12. Iyer R, Koleva S, Graham J, Ditto P, Haidt J. Understanding libertarian morality: The psychological dispositions of self-identified libertarians. PloS ONE. 2012;7(8):e42366.
