# Supplementary material for: Using moral foundations in government communication to reduce vaccine hesitancy
Source: PLoS One. 2021 Nov 9;16(11):e0259435. doi: 10.1371/journal.pone.0259435 (PMC8577733; doi:10.1371/journal.pone.0259435)
Supplement: S1 Table — Immunisation coverage in the Netherlands for the vaccinations of the National Immunisation Programme. (ZIP) [file pone.0259435.s002.zip › S1_Table.pdf]

Immunisation coverage in the Netherlands (in %)

	Newborns[1]								Toddlers[1]		Schoolchildren[1]			Adolescent Girls[1]	
Year	Cohort	DTaP-IPV	Hib	HBVa[2]	PCV[3]	MenC	MMR[4]	Full	Cohort	DTaP-IPV	Cohort	DT-IPV	MMR[4]	Cohort	HPV
2011[5]	2008	95.4	96	19.4	94.8	95.9	95.9	–	2005	92	2000	92.2	92.1	-	–
2012[6]	2009	95.4	96	19.5	94.8	95.9	95.9	–	2006	92.3	2001	93	92.6	1997	56
2013	2010	95.5	96.1	19.7	95.1	96	96.1	–	2007	92.3	2002	93.1	92.9	1998	58.1
2014	2011	95.4	95.9	51.4	95	95.8	96	–	2008	94.4	2003	92.7	92.4	1999	58.9
2015	2012	94.8	95.4	94.5	94.4	95.3	95.5	–	2009	94.1	2004	92.7	92.7	2000	61
2016	2013	94.2	94.9	93.8	93.8	94.6	94.8	93.1	2010	93.7	2005	92	92	2001	61
2017	2014	93.5	94.2	93.1	93.6	93.5	93.8	91.2	2011	93.2	2006	90.8	90.9	2002	53.4
2018	2015	92.6	93.4	92.2	92.8	92.6	92.9	90.2	2012	92.7	2007	90	90.1	2003	45.5
2019	2016	92.4	93.1	92	92.6	92.6	92.9	90.2	2013	92.5	2008	89.5	89.5	2004	45.5

[1] Immunisation coverage is assessed at the ages of 2 years (newborns), 5 years (toddlers), 10 years (schoolchildren) and 14 years (adolescent girls);
[2] HBVa only risk groups were vaccinated before 2011, from 2011 onward, universal Hepatitis B vaccination was introduced;
[3] PCV vaccine was introduced on June 1, 2006, for children born after April 1, 2006;
[4] MMR consists of two MMR vaccinations;
[5] In 2011, universal Hepatitis B vaccine was introduced;
[6] 2012 HPV vaccine was introduced

DT – Diphteria, Tetanus; aP – Pertussis (whooping cough); IPV – Poliomyelitis (polio); Hib – Haemophilus influenzae type B; HBVa – Hepatitis B; PCV – Pneumococcal disease; MMR – Mumps, Measles, Rubella; MenACWY – Meningococcal ACWY disease; MenC – Meningococcus C; HPV – Human papillomavirus
