# Supplementary material for: Using moral foundations in government communication to reduce vaccine hesitancy
Source: PLoS One. 2021 Nov 9;16(11):e0259435. doi: 10.1371/journal.pone.0259435 (PMC8577733; doi:10.1371/journal.pone.0259435)
Supplement: Attachment — Submitted filename: Response_Letter2.pdf [file pone.0259435.s005.pdf]

Response to Reviewers

Reviewer 1

Thank you for your kind interest in our study and for the helpful feedback throughout the review process! Please find in the following a brief overview of how we addressed each of your comments.

Comment 1 In the subsection "The Dutch National Immunisation Programme" (p. 5), the authors state "Our results confirm this trend, providing..." (L. 103). Is it from the current study? If so, is it appropriate to show a part of the results here in the method section?

Response 1 We agree that this sentence is somewhat premature at this particular position in the manuscript. We present our empirical results concerning the time trend for vaccination acceptance in the Results Section on page 17, which we agree is a better position for this discussion. We propose to delete the sentence you refer to on page 5.

Comment 2 Now that the authors clarify how they made the Dutch version of the MFD, I suggest the authors mention the translation issue as a limitation.

Response 2 We now mention this as limitation both in the Methods Section and in the Discussion and Conclusion.

Comment 3 In the Discussion and Conclusion section, the authors argue a possible difference between Authority and Liberty foundations. I would like to see some articles discussing the relationships between those (or other) foundations (if any). It can help readers to deeply speculate the results from the current study and moral foundations as a whole for future research.

Response 3 Thank you for proposing this valuable addition to the manuscript, which we gladly include.

Comment 4 Thank you for showing how manuscripts are structured in your field (Response 7). It helps me.

Response 4 We are glad we could clarify this issue. We would like to reiterate that we are happy to adapt this editorial aspect of our article in an eventual typesetting phase, if the editor feels this would better fit PLOS ONE's publication culture.

Reviewer 2

Thank you for your kind interest in our study and for the helpful feedback throughout the review process! Please find in the following a brief overview of how we addressed each of your comments.

Comment 1 Table 2 lists 26 brochures. Please highlight the brochures which were not considered for analysis in the table itself to avoid the confusion.

Response 1 We now recognise how the representation of Table 2 seems to have caused confusion. Thank you for reiterating this issue to bring it to our attention again. As some brochures have comparably long names (i.e. "Folder baby's van 2, 3, 4 en 11 maanden 2011"), there is a line break in the particular table cell. We now apply alternating light-grey row colours to tell different brochures from mere line breaks.

Comment 2 The paper needs serious proof-reading. There are several grammatical mistakes and other errors. e.g. the reference to subsections are missing at many places : pg 17-line 338, pg 10- line 222, page 10-line 224.

Response 2 We have performed another round of proofreading and adjusted a number of formulations which we hope elevates the language use within our manuscript to an acceptable level. We apologise for the dead links/references to some of the sections and subsections which we caused by changing the associated command structure within our typesetting software. We hope this issue has been resolved now.

Comment 3 As mentioned in pg 17, line 335, fairness/cheating has been discarded from analysis. FC should also be removed from Equation 1.

Response 3 Thank you for pointing this out to us. We agree that this dimension may be discarded from Equation 1. For consistency sake we also dropped it from our regression outputs in Tables 5 and 10.

Comment 4 Since MF dimensions do not vary spatially. I do not see the point of analysing it at national, regional and municipal levels. It is rather surprising that the coeffs for MF dimensions vary significantly at the different spatial resolutions since the MF dimensions and other control variables such as degree of difficulty (which are again derived from linguistic analysis of brochures) and time remain constant across the regions. Is it only modelling the variations in population sizes? Loyalty/betrayal effect became significant at regional and municipal level but not at the national level, however, the values of MF dimensions remain the same.

Response 4 The purpose of executing the analysis at different spatial levels is to assess the robustness of the results. Concretely, we are interested in demonstrating the results' sensitivity to different ways of measuring the same thing. We add a brief clarification on page 14.

Comment 5 OLS with time-demeaning fixed effects would suggest that the moral dimensions remain constant over time. Is this a valid assumption?

Response 5 The fixed effects panel is not the moral dimensions, which indeed vary over time. Our panel is the type of vaccination in a region. For data on the national level, this would be one of ten vaccinations (i.e. DTaP-IPV newborns, Hib, etc.). For the regional level this would be a convex combination of the ten vaccination types and 25 regions, resulting in 250 panels (equivalent for municipal data). More concretely (and ignoring the control variables for brevity sake) we try and estimate $y_{ijt} = \alpha + \boldsymbol{x}_{it}\boldsymbol{\beta} + \nu_{ij} + \epsilon_{ijt}$, where y_{ijt} is the vaccination rate for vaccination i in region j for time t. Further, let $\boldsymbol{x}_{it}$ be the set of Moral Foundations scores for i in t. ν_{ij} then is the vaccination and region specific error term; it differs between units, but for any particular unit, its value is constant. Within the Netherlands there exists some degree of regional variation as to the general vaccination acceptance, and each type of vaccination also displays some heterogeneity in vaccination rate. By demeaning these fixed effects, we are able to better isolate the true $\boldsymbol{\beta}$ without the region- or vaccination-specific fixed effects. We add a clarifying sentence on page 15.

Comment 6 Authors should not make strong claims regarding the effect of MF dimensions on vaccine hesitancy on the basis of only 22 sample points and there could be many other factors affecting the hesitancy which have not been considered for the analysis. Also, consider that the moral foundations are not completely independent of each other which is also evident from the MF dictionary words.

Response 6 The first part of this comment seems akin to comment 4 from review round 1 which discusses the issue of overfitting. The subjects in our study have no influence on which version of the brochures they receive, i.e. there is no self-selection by subjects and exposure resembles random assignment. In that sense, we would categorise our research design as a natural experiment in that clusters of subjects have been exposed to different (experimental/control) conditions determined by factors outside the control of us investigators. Among the long tradition of natural experiments, Angrist and Evans [1], for example estimate the effect of family size on the mother's labour market outcomes. Sargent et al. [2] employ a temporary smoking ban in all public spaces in Helena (Montana) to investigate its effect on the rate of heart attacks at the only local hospital. We hope that these examples from a long tradition of literature may convince the reviewer that the 22 different treatment-like realisations in our study actually constitute a comparably fine granulation when comparing with other natural experiments.

This not withstanding, we do acknowledge that vaccine hesitancy is a multi-faceted phenomenon (see, e.g. page 17) and that many different factors play a role in determining the decision whether or not to accept a vaccine. Still, we believe that the power of our study is strong enough to contribute to increasing our understanding of this decision process and some of the aspects it is significantly impacted by. In the Discussion and Conclusion Section we discuss the relationship between the MFT dimensions.

References

1. Angrist JD, Evans WN. Children and Their Parents' Labor Supply: Evidence from Exogenous Variation in Family Size. The American Economic Review. 1998;88(3):450–

477.

2. Sargent RP, Shepard RM, Glantz SA. Reduced incidence of admissions for myocardial infarction associated with public smoking ban: before and after study. BMJ. 2004;328(7446):977–980. doi:10.1136/bmj.38055.715683.55.
